# Identification of three metabolic subtypes in gastric cancer and the construction of a metabolic pathway-based risk model that predicts the overall survival of GC patients

**DOI:** 10.3389/fgene.2023.1094838

**Published:** 2023-02-10

**Authors:** Tongzuan Chen, Liqian zhao, Junbo Chen, Gaowei Jin, Qianying Huang, Ming Zhu, Ruixia Dai, Zhengxi Yuan, Junshuo Chen, Mosheng Tang, Tongke Chen, Xiaokun Lin, Weiming Ai, Liang Wu, Xiangjian Chen, Le Qin

**Affiliations:** ^1^ Department of Gastrointestinal Surgery, The First Affiliated Hospital of Wenzhou Medical University, Wenzhou, Zhejiang, China; ^2^ Department of Neurosurgery, Nanfang Hospital, Southern Medical University, Guangzhou, Guangdong, China; ^3^ School of Ophthalmology and Optometry, Eye Hospital, Wenzhou Medical University, Wenzhou, Zhejiang, China; ^4^ Second School of Clinical Medicine, Wenzhou Medical University, Wenzhou, Zhejiang, China; ^5^ College of International Education, Henan University, Kaifeng, Henan, China; ^6^ Scientific Research Laboratory, Lishui City People’s Hospital, Lishui, Zhejiang, China; ^7^ Laboratory Animal Centre, Wenzhou Medical University, Wenzhou, Zhejiang, China; ^8^ The Second Affiliated Hospital and Yuying Children’s Hospital of Wenzhou Medical University, Wenzhou, Zhejiang, China; ^9^ Department of Pathology, The First Affiliated Hospital of Wenzhou Medical University, Wenzhou, Zhejiang, China; ^10^ Department of Pediatric Surgery, The Second Affiliated Hospital and Yuying Children’s Hospital of Wenzhou Medical University, Wenzhou, Zhejiang, China

**Keywords:** gastric cancer, prognosis, metabolism, molecular subtypes, multi-omics

## Abstract

Gastric cancer (GC) is highly heterogeneous and GC patients have low overall survival rates. It is also challenging to predict the prognosis of GC patients. This is partly because little is known about the prognosis-related metabolic pathways in this disease. Hence, our objective was to identify GC subtypes and genes related to prognosis, based on changes in the activity of core metabolic pathways in GC tumor samples. Differences in the activity of metabolic pathways in GC patients were analyzed using Gene Set Variation Analysis (GSVA), leading to the identification of three clinical subtypes by non-negative matrix factorization (NMF). Based on our analysis, subtype 1 showed the best prognosis while subtype 3 exhibited the worst prognosis. Interestingly, we observed marked differences in gene expression between the three subtypes, through which we identified a new evolutionary driver gene, CNBD1. Furthermore, we used 11 metabolism-associated genes identified by LASSO and random forest algorithms to construct a prognostic model and verified our results using qRT-PCR (five matched clinical tissues of GC patients). This model was found to be both effective and robust in the GSE84437 and GSE26253 cohorts, and the results from multivariate Cox regression analyses confirmed that the 11-gene signature was an independent prognostic predictor (*p* < 0.0001, HR = 2.8, 95% CI 2.1–3.7). The signature was found to be relevant to the infiltration of tumor-associated immune cells. In conclusion, our work identified significant GC prognosis-related metabolic pathways in different GC subtypes and provided new insights into GC-subtype prognostic assessment.

## Introduction

Gastric cancer (GC) is a common malignancy and is considered a main contributor to cancer-related deaths throughout the world (Erratum, 2020). GC classification is typically done *via* the Lauren or WHO classifications, as well as the TNM grading system, according to the clinicopathologic characteristics. This, to a large degree, dictates appropriate therapy (Ajani et al., 2016; Daum et al., 2020). GC is a heterogeneous disease (Chia and Tan, 2016). Tumor heterogeneity indicates the presence of differences between patients with one type of malignancy or among the tumor cells of one patient, with variations in both genotype and phenotype. As such, patient prognosis can vary significantly, even when patients present with similar clinical characteristics and receive similar treatments. This typically signifies that the use of clinicopathologic factors and the current classification systems are ineffective for accurate prognosis prediction and risk-stratified analyses (Shah and Ajani, 2010; Noh et al., 2014). Hence, it is of great importance to identify new signatures, with higher accuracy of prediction, to improve the prognosis of GC patients.

Metabolic reprogramming is a major feature of solid tumors, and it contributes directly to the malignancy of tumors through the processes of metastasis, invasion, and disease progression (Boroughs and DeBerardinis, 2015; Sun et al., 2018). As described above, GC tissues are highly heterogeneous. This applies also to metabolic heterogeneity, which represents metabolic heterogeneity and flexibility between tumors or even different regions of the same solid tumor (Sathe et al., 2020; Sexton et al., 2020). Furthermore, tumor metabolites can reshape the tumor microenvironment (TME) mediated by various metabolic signaling pathways that promote tumorigenesis (Jiang et al., 2019; Xiang et al., 2019). Brand et al. (2016) discovered that LDHA-derived lactate reduced the ability of T cells and NK cells to conduct immune surveillance of cancer cells. Furthermore, Leone et al. (2019) reported the activation of different metabolic pathways through the blockage of glutamine in cancer cells to overcome immune invasion. Increasing evidence highlights the profound significance of metabolic heterogeneity in GC, which may explain the challenges faced by current therapeutics and in the prediction of clinical outcomes of GC patients (Zhang et al., 2020a; Zhu et al., 2021a). Given that there is still much to learn about the metabolic heterogeneity of GC, it is critical to explore and identify new potential metabolic signatures related to GC prognosis.

In our research, we established subtypes of GC based on genes associated with a number of core metabolic pathways, including, but not limited to, glycolysis, nucleic acid metabolism, amino acid metabolism, and lipid metabolism in tumors, using normalized RNA-Seq data from the public databases *Gene Expression Omnibus* (GEO) and *The Cancer Genome Atlas* (TCGA). We further analyzed the genomic distinctions of these molecular isoforms to assess their potential roles in prognosis prediction. To achieve this, we generated a model of risk prognostication using differentially expressed genes (DEGs) between the different GC forms, with verification of selected DEGs in clinical samples using qRT-PCR. Moreover, we explored the relationship between immune cell infiltration and our newly developed model and analyzed further biological characteristics and functional enrichment of the DEGs. Lastly, the stability of the risk model was validated using an external database. In summary, we analyzed the metabolic features of GC, integrating these with the current clinicopathologic characteristics and grading system for GC. A predictive model based on specific genetic features was developed, and the incorporated genes may serve as potential therapeutic targets for GC as well for accurate prediction of patient outcomes.

## Methods

### Data sources and preprocessing

We collected the original gene expression profiling data of tumor and adjacent normal tissues, as well as the clinical characteristics of GC cohorts from the Gene Expression Omnibus (http://www.ncbi.nlm. nih.gov/geo/) database (Barrett et al., 2013). After the removal of samples without complete survival information, 865 GC samples and patient information were included in the analysis. This included patients from the GSE84437 and GSE26253 cohorts. We used the 865 GEO GC study cohort with 865 patient information for validation of our prognostic model. The GC RNA-Seq data, as well as corresponding clinical data (415 tumor and 34 para-tumor samples), were extracted from The Cancer Genome Atlas (TCGA, https://portal.gdc.cancer.gov/) website (Wang et al., 2016). The format of the downloaded data was HTSeq-Counts and was normalized and preprocessed using uniform log_2_ (exprset+1). A filtered sample of 350 patients was included in the study. The TCGA data filtering criteria were as follows: 1. complete prognostic information; 2. survival time > 1 month; 3. patients with both GC and healthy volunteer transcriptome data; 4. removal of duplicate and normal samples. Genomics data were also obtained from the TCGA database, and after the removal of unmatched patients, the genomic data of 348 patients were included in our final analysis. Table 1 summarizes the clinical baseline information of all cohorts used in this study.

**TABLE 1:** Clinical baseline information of patients from TCGA and GEO.

Characteristic	TCGA (*n* = 350)	GSE84437 (*n* = 433)	GSE26253 (*n* = 432)
Age (%)			
<=65	6	283	NA
>65	344	150	NA
Sex (%)			
Female	124	137	NA
Male	226	296	NA
Race (%)			
Asian	72	NA	NA
Black	10	NA	NA
White	222	NA	NA
Other	46	NA	NA
Tumor_grade (%)			
G1	9	NA	NA
G2	125	NA	NA
G3	207	NA	NA
G4	9	NA	NA
Pathologic_stage (%)			
T1	16	NA	68
T2	74	NA	167
T3	161	NA	130
T4	95	NA	67
Stage_T (%)			
T1	16	11	NA
T2	74	38	NA
T3	161	92	NA
T4	95	292	NA
TX	4	0	NA
Stage_M (%)			
M0	312	NA	NA
M1	23	NA	NA
MX	15	NA	NA
Stage_N (%)			
N0	103	80	NA
N1	93	188	NA
N2	72	132	NA
N3	71	33	NA
NX	11	0	NA
Status (%)			
Alive	204	224	255
Dead	146	209	177

Moreover, the single-cell transcriptome dataset GSE184198 was selected from the GEO Datasets of NCBI, and the tumor sample GSM5580154 was extracted. The single-cell transcriptome data were processed using the Seurat package. Firstly, the single-cell data were subjected to cell quality control and low-quality cells were filtered out. Cells with ribosomal gene expression percentage greater than 20% and red blood cell gene expression percentage greater than 3% were removed, and 9,383 cells that met the criteria were screened out. The NormalizeData function was used to normalize the single cell data, the FindVariableFeatures function was used to find hypervariable genes, and the ScaleData function was used to normalize the hypervariable genes. The principal component analysis (PCA) was performed using the hypervariable genes. Cells were clustered by the FindNeighbors function and FindClusters function. The annotation results were divided into epithelial cells, B cells, T cells and other cell types. Using the scMetabolism package, each cell was scored using the VISION algorithm to derive activity scores for different cell types in different metabolic pathways (Wu et al., 2022).

### Acquisition of metabolism-related genes

Metabolism-related genes were identified from the Molecular Signatures Database (MSigDB) (https://www.gsea-msigdb.org/gsea/msigdb/index.jsp) (Subramanian et al., 2005). Overall, 12 metabolic gene sets were obtained, which included the reactome pyrimidine catabolism, reactome pentose phosphate pathway, reactome purine catabolism, reactome metabolism of amino acids and derivatives, reactome citric acid/TCA cycle and respiratory electron transport, reactome glycogen metabolism, reactome metabolism of lipids, reactome fatty acid metabolism, reactome glutamate and glutamine metabolism, reactome pyruvate metabolism, reactome glucose metabolism, and reactome metabolism of nucleotides.

### Gene set variation analysis and functional enrichment analysis

Evaluation of the relative enrichment of select genomes was conducted using Gene set variation analysis (GSVA), which is generally employed to reflect pathway variation across a sample population (Hanzelmann et al., 2013). Functional enrichment and pathway analyses, which cluster genes with similar functions and correlate them with biological phenotypes, were executed with the R package “cluster Profiler” (Yu et al., 2012).

### Identification of DEGs

The “limma” package in R was used to examine DEGs between tumors and adjacent normal tissues (Ritchie et al., 2015). The DEG filter threshold was set at |log2 FC (fold-change)| >1 and *p* < 0.05. In order to obtain more DEGs, we relaxed the DEG screening standards, i.e., we chose the pre-correction *p*-value, because we could tolerate the problem of excessive false positives, since this evaluation would be followed up with more stringent gene screening criteria, such as, univariate cox regression, random forest, and lasso regression to identify the most critical DEG metabolism-related genes.

### Evaluation of immune cell infiltration

The “Cell-type Identification By Estimating Relative Subsets Of RNA Transcripts (CIBERSORT)” (Newman et al., 2015) (https://cibersort.stanford.edu) algorithm was used to predict immune cell infiltration in the GC samples. After entering the expression data of the samples, the proportions of 22 types of infiltrating immune cells were obtained. CIBERSORT was used to calculate the *p*-value, which was dependent on the Monte Carlo permutation test. Lastly, *p* < 0.05 was considered significant.

### Consensus molecular clustering of 1,489 core metabolic regulators by NMF

We next assessed the 12 metabolic gene sets in MSigDB, including reactome pyrimidine catabolism, reactome pentose phosphate pathway, reactome purine catabolism, reactome metabolism of amino acids and derivatives, reactome citric acid/TCA cycle and respiratory electron transport, reactome glycogen metabolism, reactome metabolism of lipids, reactome fatty acid metabolism, reactome glutamate and glutamine metabolism, reactome pyruvate metabolism, reactome glucose metabolism, and reactome metabolism of nucleotides. After merging of the genes and removing duplicates, a total of 1,489 metabolism-related genes were selected. We conducted consensus clustering using NMF to identify different metabolic preference patterns, based on the expression of 1,489 regulators. The expression of 1,489 metabolic regulators [Matrix V, Gene (F) × Patient (N): 1,489 × 350] was factorized into 2 non-negative matrices W [Gene (F) × Patient program (K): 1,489 × 3] and H [Expression program (K) × Patient (N): 3 × 350] (i.e., V ≈ WH). Specifically, we decompose the matrix V into a basis matrix W and a coefficient matrix H. On the one hand, the basis matrix W is characterized by patient programs: each one of patient programs is a vector in W. On the other hand, each column vector of the coefficient matrix H can be regarded as the coordinates obtained by projecting the corresponding column vector of the matrix V onto W: each expression program is a row in H. Repeated factorization of matrix V was performed, and its outputs were aggregated to obtain consensus clustering of GC samples. The optimal number of clusters was then selected, according to the cophenetic, dispersion, and silhouette coefficients. The R package ‘NMF’ (version 0.23.0) with the brunet algorithm and 100 n runs were used to perform the consensus clustering.

### Identification of the 11-gene signature

To further identify prognosis-related genes in GC, we initially screened 412 significant genes by univariate Cox proportional-hazards regression analysis of NMF_corDEGs (1841 DEGs among 3 metabolic subtypes) using the “coxph” function in the “survival” R package. To eliminate multicollinearity among these candidate genes, we applied LASSO regression with optimal penalty parameters and minimum 10-fold cross-validation to screen 30 independent prognosis-related genes. Subsequently, the “randomForestSRC” R package was used to reduce the size of candidate genes based on their variable importance (VIMP) and minimum depth (Ishwaran et al., 2014). Only genes with VIMP > 0.01 along with their corresponding minimum depth were selected, and a total of 26 genes were obtained after intersection with the LASSO regression results. After further adjustment, multivariate Cox regression (stepwise model) was performed to identify the pivotal genes, and finally, 11 gene signatures were obtained. At this point, glmnet package is used for lasso regression analysis, and My stepwise package is used for Cox model building The coefficients obtained from the regression algorithm were used to obtain the risk scores based on the following formula: riskscore = valGene1*β1 + valGene2*β2 + ⋯+ valGenen*βn. Furthermore, according to the above formula, the risk scores of GC patients were calculated separately, and the patients were divided into high-risk and low-risk groups using the median as the cut-off value (Sullivan et al., 2004).

### Clinical patient sample collection and gene expression verification

Five matched GC tissue samples from patients who were diagnosed at the Department of Gastrointestinal Surgery of the First Affiliated Hospital of Wenzhou Medical University were obtained between October 2021 and January 2022. The distance between the tumor tissue and adjacent normal tissue was > 5 cm. This study received approval from the Institutional Ethics Committees of the First Affiliated Hospital of Wenzhou Medical University and followed the guidelines of the Declaration of Helsinki. The ethics ID number related to experiments in this study is 2019–089. All participants were fully informed and signed informed consent forms prior to participating in this research. Total RNAs from the tumor and adjacent tissues of GC patients were extracted using TRIzol reagent (Invitrogen). The cDNA synthesis was done *via* Taq DNA Polymerase (Bio-Rad) and the qRT-PCR was performed on the CFX96 optics module connected to a C1000 thermocycler system (Bio-Rad). β-actin was used as internal control, and fold change (2^−△△CT^) was used to express the relative gene expression. The primer sequences are listed in Supplementary Table S1.

### Statistical analysis

R software (version 4.0.1) was used for all statistical analyses, and the χ^2^ test was used for analysis of differences between two groups of categorical variables. Student’s *t*-test or Wilcoxon rank-sum test was used for conducting differential comparisons. One-way analysis of variance or the Kruskal–Wallis test was used to conduct differential comparisons of multiple groups. The Kaplan–Meier method and the log-rank test were used for survival analyses. *p* < 0.05 was considered statistically significant.

## Results

### Identification of molecular isoform-based metabolic signatures in GC, based on NMF

**FIGURE 1:**
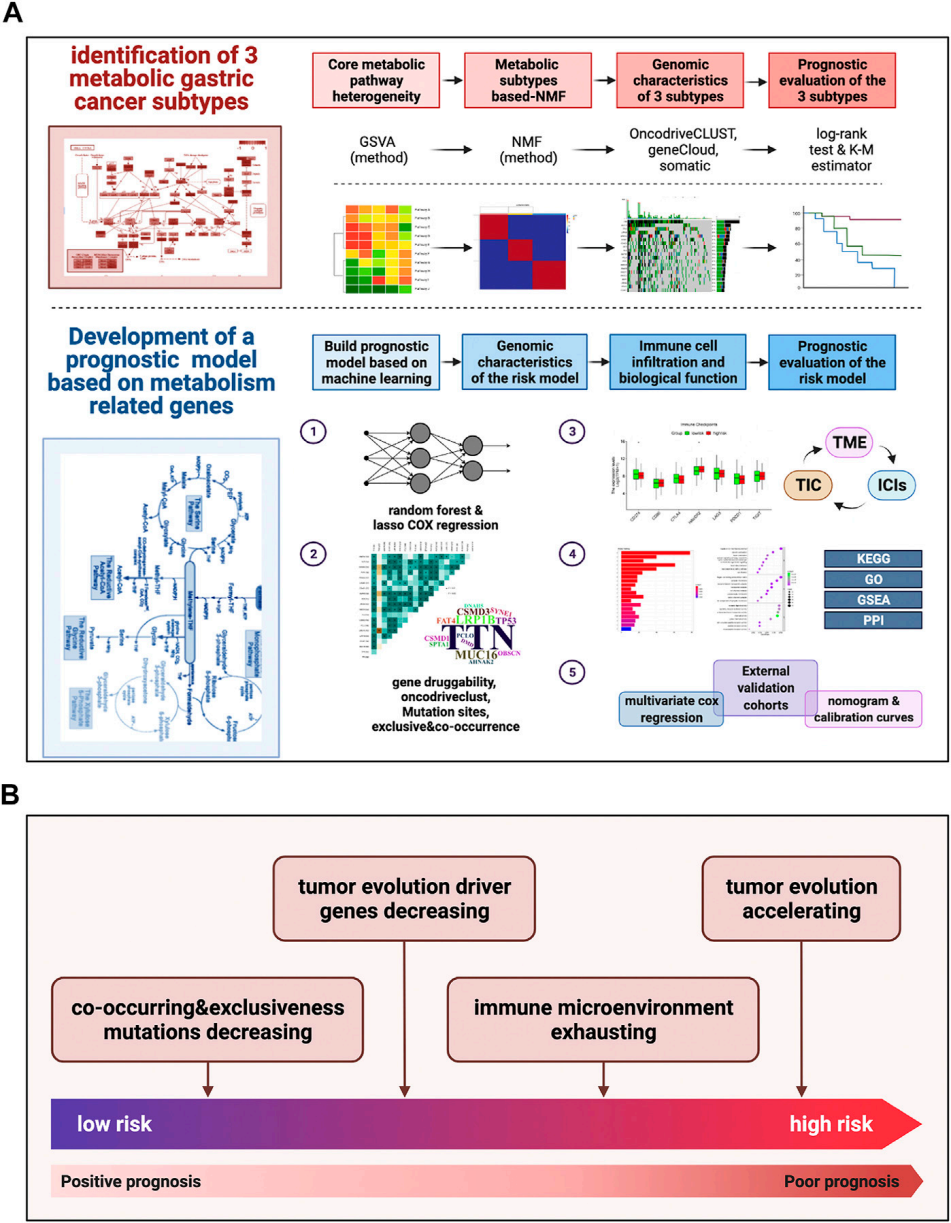
Pipeline of the study design and a summary of main work and findings **(A)** Our detailed pipeline of the study design. **(B)** A summary of our main work and findings.

**FIGURE 2:**
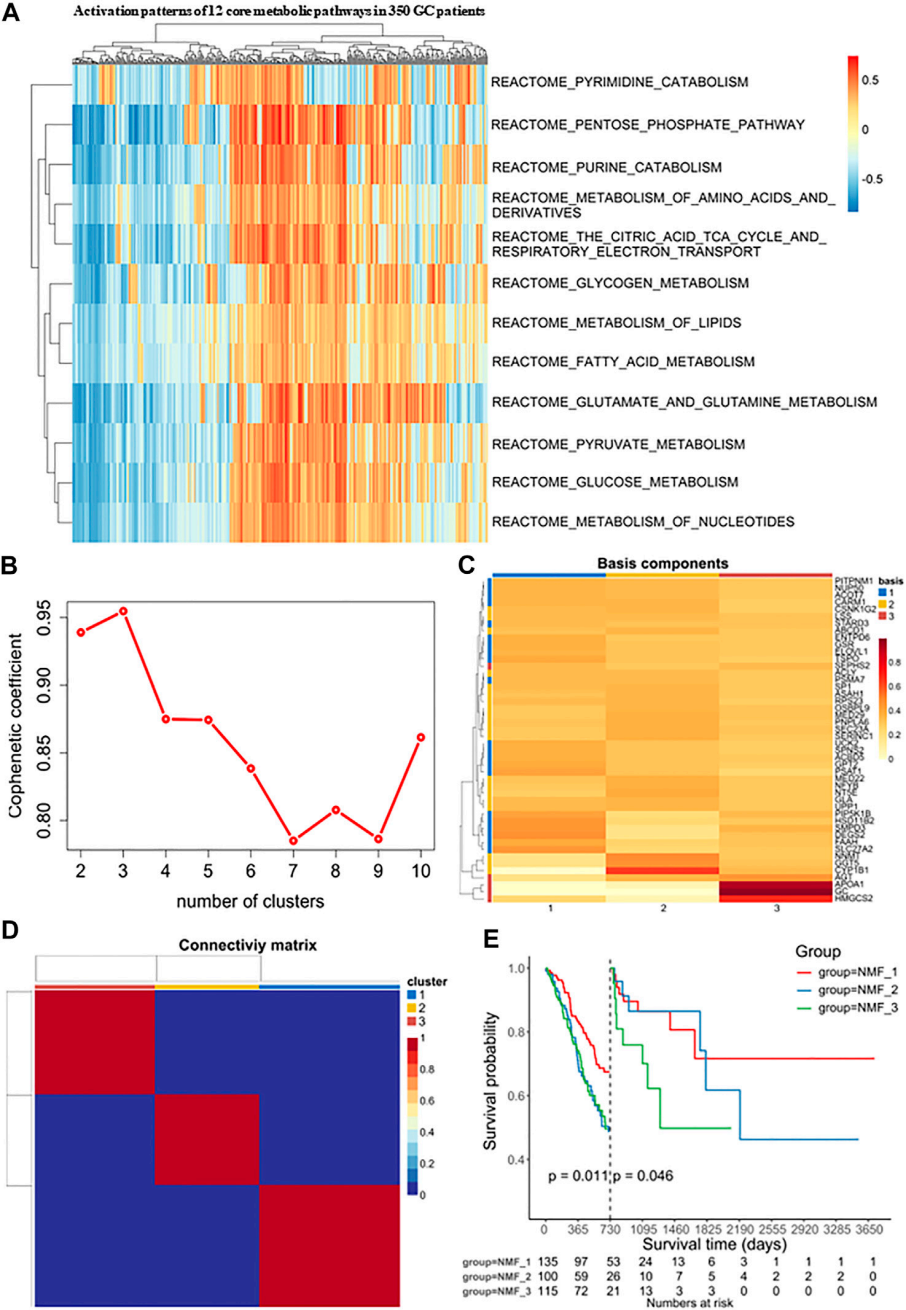
Identification of molecular isoforms for metabolic signatures of gastric cancer based on NMF **(A)** Heatmap of enrichment score of 12 main metabolic pathways gene sets in GC and adjacent non-tumor samples, which demonstrated metabolic heterogeneity among patients with gastric cancer. **(B)** The optimal number of clusters was selected with factoextra package. **(C)** Classification of expression profiles into 3 categories based on signature levels using NMF. **(D)** GC samples were clustered by non-negative matrix factorization (NMF) method. **(E)** Survival analysis was used to evaluate the different survival patterns between metabolic subtypes.

The detailed pipeline of the study design is illustrated in Figure 1A, and a summary of the main work and findings is shown in Figure 1B. First, we investigated the activation and deactivation of 12 major metabolic pathways in GC samples using GSVA. The 12 major pathways included the reactome pyrimidine catabolism, reactome pentose phosphate pathway, reactome purine catabolism, reactome metabolism of amino acids and derivatives, reactome citric acid/TCA cycle and respiratory electron transport, reactome glycogen metabolism, reactome lipid metabolism, reactome fatty acid metabolism, reactome glutamate and glutamine metabolism, reactome pyruvate metabolism, reactome glucose metabolism, and reactome nucleotide metabolism (Figure 2A). In addition, metabolic analysis of these 12 pathways in the single-cell dataset showed that most metabolic pathways were enriched in epithelial cell types (Supplementary Figure S1D, E). These highlight the presence of marked metabolic heterogeneity in GC samples.

Next, Consensus Cluster Plus was used to identify a variety of genes derived from the above pathways. At present, this algorithm is widely used in cancer research as a commonly used analysis method in NMF typing and can be used to determine the optimal number of clusters K. In our study, the clustering was deemed to be most robust at k = 3, where the steepest drop-off of the cophenetic correlation coefficients appeared (Figures 2B, C). The expression levels of these metabolism-related genes in the three GC subtypes is shown in a heatmap (Figure 2D). The three subtypes were distinguished using principal component analysis (PCA) and clustering (Supplementary Figure S1A). Additionally, it was clear that the prognoses associated with these subtypes were distinct. In the NMF_3 subtype, patient prognosis ranked the worst after two years (*p* = 0.046, Figure 2E) while the NMF_1 subtype showed the best outcomes after two years (*p* = 0.011, Figure 2E), with the NMF_2 group falling in between NMF_3 and NMF_1. To ensure the reproducibility of the Consensus Cluster Plus results, we validated the results using two external datasets, namely, GSE66229 and GSE26253. In the GSE66229 cohort, GC patients were also found to be clustered into three categories, and the prognosis of the three subtypes was significantly different (Supplementary Figure S1B-1, 2, 3). In the GSE26253 cohort, the Kaplan-Meier curves of the three subtypes showed slight differences in survival outcomes, although the differences were non-significant possibly due to insufficient sample size or the high heterogeneity of GC (Supplementary Figure S1C-1, 2, 3).

### Comparing the inter-genomic differences between GC molecular subtypes

**FIGURE 3:**
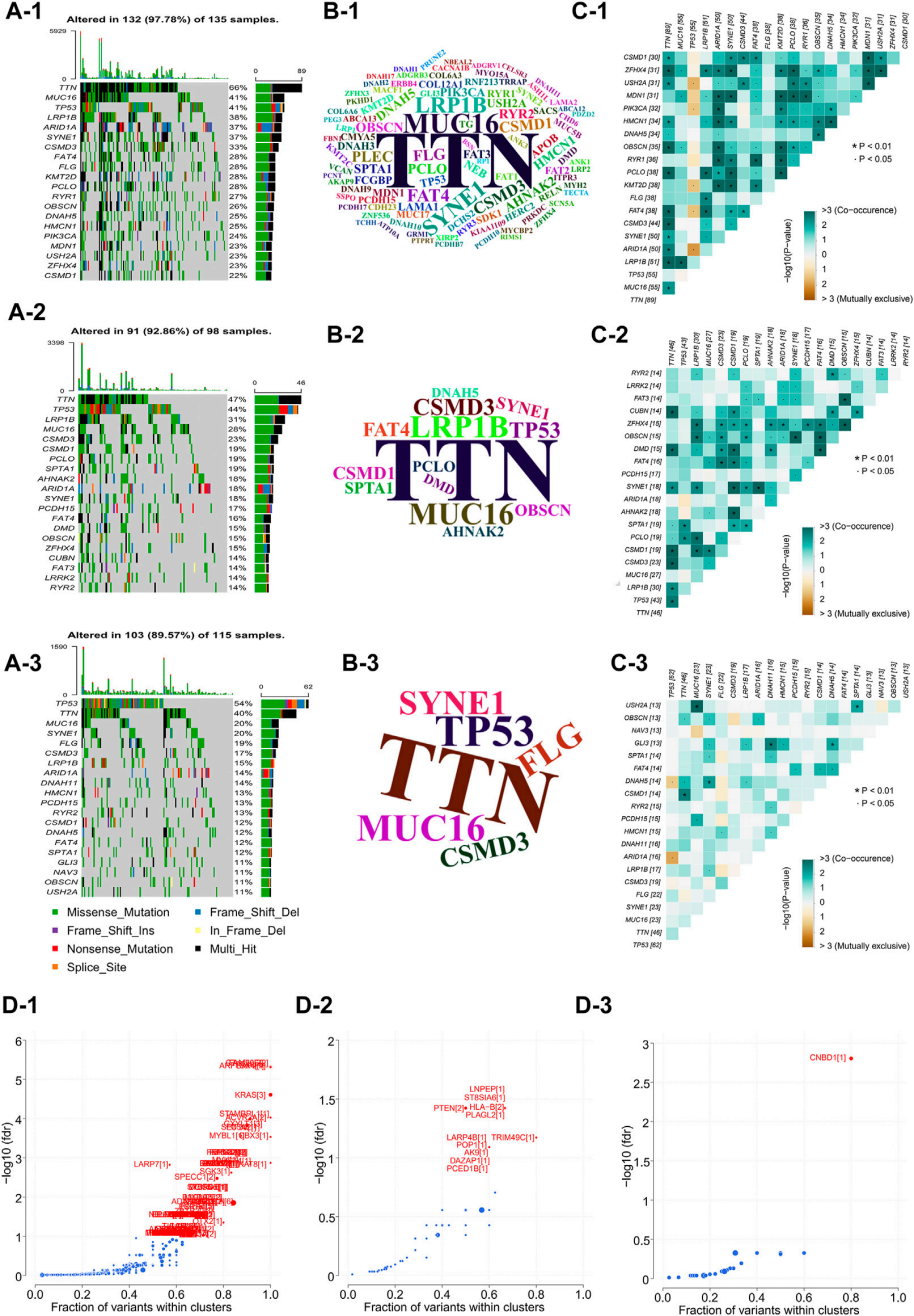
Comparison of inter-genomic differences between molecular isoforms **(A)** Waterfall plot of the top 20 mutant genes in the 3 metabolic subtypes (A-1: NMF_1, A-2: NMF_2, A-3: NMF_3). **(B)** Wordclouds were drawn with size of scripts bound up with amounts of samples containing certain mutant genes in the 3 metabolic subtypes (B-1: NMF_1, B-2: NMF_2, B-3: NMF_3). **(C)** Correlation heat map to show the degree of correlation between mutant genes in the 3 metabolic subtypes, suggesting that the worse the typing prognosis, the less correlated the mutation is (C-1: NMF_1, C-2: NMF_2, C-3: NMF_3). **(D)** Volcano map used to demonstrate mutation driver genes in the 3 metabolic subtypes (D-1: NMF_1, D-2: NMF_2, D-3: NMF_3).

In order to analyze and visualize mutation(s) in somatic cells of the three GC subtypes, we utilized the R package “maftools”. The genomic data of 348 patients were analyzed, including those of 135 NMF_1 patients, 98 NMF_2 patients, and 115 NMF_3 patients. As depicted in the waterfall plot in Figure 3A, different colors were used to distinguish between different types of mutations and show detailed information on the mutants in each sample. The results showed that the top three mutated genes in patients in the NMF_1 subtype were *TTN* (66%), *MUC16* (41%), and *TP53* (41%), while those in NMF_2 patients were *TTN* (47%), *TP53* (44%), and *LRP1B* (31%), and those in NMF_3 were *TP53* (54%), *TTN* (40%), and *MUC16* (20%). These results also demonstrated that in the three groups, GC patient prognosis was negatively correlated with increased incidence of mutated *TP53* and positively correlated with increased mutations in *TTN* and *MUC16*. Meanwhile, word clouds were drawn with the size of scripts reflecting the numbers of mutant genes in the samples (Figure 3B). It was apparent that there were a greater number of mutations in the NMF_1-subtype were counted the most while, while NMF_3 had the fewest. Interestingly, patients carrying more mutations had a more favorable prognosis while those with fewer mutations tended to have unfavorable outcomes. This finding is consistent with the results of a report from JAMA Oncology, which concluded that *MUC16* mutations were associated with increased numbers of mutations in tumors together with better survival outcomes in GC patients (Li et al., 2018). In addition, the correlations between somatic cell mutations in the different groups were analyzed. This showed that somatic cells of the NMF_1-subtype showed the highest incidence of chain mutation and unique mutations while the incidence of common and unique mutations was lowest in the NMF_3 subtype (Figure 3C). These data suggest the potential for analyzing mutations and their expression in developing treatments for GC. At this point, we inferred that the ratio of subtype-unique mutations to mutations in *TP53*, involving genes such as *USH2A*, *PIK3CA*, *KMT2D*, *FAT4*, and *ARID1A*, was positively related to patient prognosis. To confirm this, we performed a more comprehensive analysis with patient prognostic information. Associations between the most highly mutated genes in the three subtypes were then investigated. This identified three genes that were exclusively correlated with mutations in p53, namely, PIK3CA, KMT2D, and ARID1A (Supplementary Figure S2A). As expected, these three genes were positively correlated with GC patient prognosis, i.e., the higher the gene expression, the better the patient prognosis (Supplementary Figures S2B–D). In addition, we further explored the gene PIK3CA that was exclusively associated with TP53 mutations and occurred only in subtype 1 (suggesting an association with good prognosis). The GC patients were divided into four groups, namely, TP53-/PIK3CA-, TP53-/PIK3CA+, TP53+/PIK3CA-, and TP53+/PIK3CA+, and Kaplan-Meier analyses were conducted to estimate the prognostic impact of TP53 and PIK3CA mutations. This revealed that the patients in the TP53-/PIK3CA + group had better outcomes compared with the TP53+/PIK3CA-group, although the difference was non-significant on the log-rank test (Supplementary Figure S2E). This may have been due to insufficient sample size. In addition, we also identified the most common mutated gene in GC, *MUC16*, which showed a potentially exclusive mutational relationship with LRP1B. Hence, we divided the patients into the MUC16-/LRP1B+ and MUC16+/LRP1B- groups and performed survival analysis which showed that the former group experienced worse outcomes (Supplementary Figure S2F, log-rank test, *p* = 0.066). We also explored driver genes that drove mutations in the three subtypes and identified a new driver gene termed *CNBD1* (Figure 3D). Interestingly, only a single evolutionary driver gene CNBD1 was present in the NMF_3 subtype with the worst outcomes. This was relevant to the malignancy and prognosis of GC. Survival analysis and log-rank tests were performed for CNBD1, showing that it was, indeed, associated with worse outcomes in patients with GC (Supplementary Figure S2G, *p* = 3.5e−05, HR = 1.62, 95%CI 1.28–2.03, *n* = 631). The result was also primarily intended to show that outcomes were ranked as NMF1 > NMF2 > NMF3, and that the evolutionary driver genes for the three subtypes also tended to vary from more to less in this process (Figure 3D). This observation was very interesting and suggested that the worse the prognosis of the patient, the greater the evolutionary pressure, and, therefore, the less the expression of the evolutionary driver genes. Furthermore, we carried out analyses on the druggability of the mutant genes in these subtypes, as well as the crosstalk between genes and drugs, thus displaying the latent classification of druggable genes (Supplementary Figure S2H). The identification of the evolutionary driver genes was mainly based on the Oncodrive CLUST (Tamborero et al., 2013).

### Construction of a prognostic risk model based on metabolism-related genes

**FIGURE 4:**
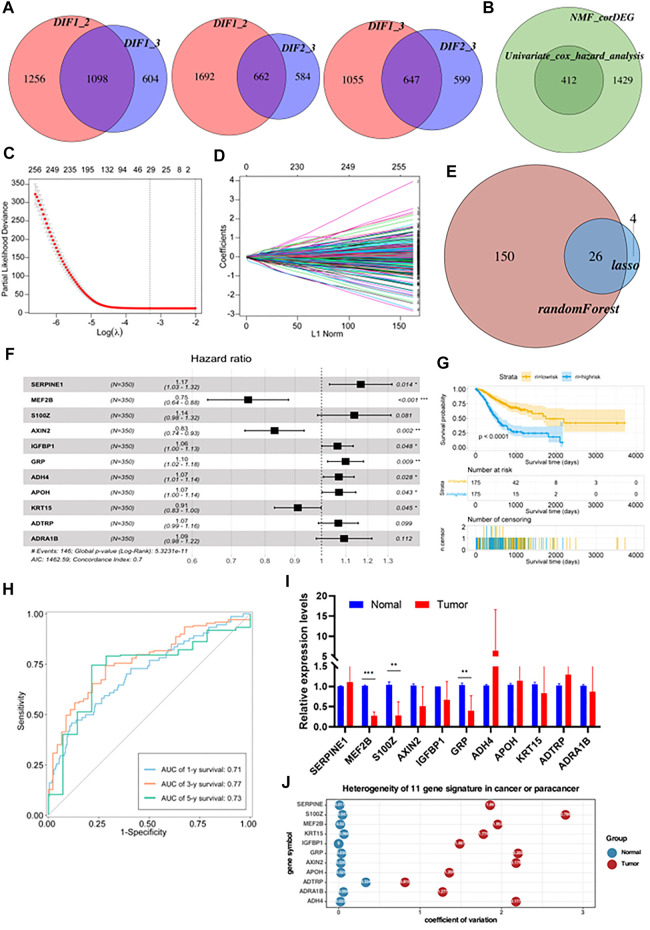
Construction of a prognostic risk model based on metabolism-related genes **(A)** The differential enrichment score of gene sets was calculated between each of the two subgroups and intersected them. Subtype 1, subtype 2 and subtype 3 had 1,098, 662 and 647 distinct gene sets, respectively. **(B)** Batch Cox regression analyses screening for prognosis-related differential genes. **(C)** The dotted vertical lines represent the optimal values of λ. The top *x*-axis has the numbers of gene sets, whereas the lower *x*-axis revealed the log (λ). **(D)** Least absolute shrinkage and selection operator (LASSO) coefficient profiles (*y*-axis) of the gene sets and the optimal penalization coefficient (λ) *via* 10-fold cross-validation based on partial likelihood deviance. **(E)** Take the intersecting genes after lasso and random-forest screening. **(F)** Constructing a stepwise Cox proportional hazards model. **(G)** Kaplan–Meier OS curves with difference detection by log-rank test for patients from the training datasets. **(H)** ROC curve analyses based on the 11 gene signature. **(I)** Relative expression levels of mRNA for 11 gene signature. **(J)** Coefficient of variation of 11 gene signature in cancer and paracancerous tissues.

Using the limma package in R with a threshold of *p* < 0.05 and absolute of |log_2_ FC| > 2, intersections in the expression of DEGs between two of the three subtypes were obtained (Figure 4A). This identified 1841 common DEGs which were analyzed by univariate Cox regression analysis to select meaningful genes to generate models (Figure 4B). Next, the glmnet package was used to conduct LASSO-Cox regression analysis. As shown in Figure 4C, the plot depicts the tracks of each independent variable. The results revealed that with a decrease in λ (lambda value), the number of coefficients of the independent variable close to 0 increased. We next used a 10-fold cross-validation to determine the best λ, and we observed that the regression coefficients of most of the variables stabilized when the λ was around −3.324. Hence, it was considered that the λ can be set to −3.324. Using this parameter, 29 genes were selected for subsequent analyses. The confidence intervals of each λ are displayed in Figure 4D. To identify genes with high prognostic importance in GC, we employed the randomForest R package to include prognostically relevant DEGs in the model, identifying 176 genes with high prognostic impact from 412 genes, and the intersection of these with the results of the LASSO regression was determined (Figure 4E). Additionally, stepwise multivariate Cox regression was used to further refine the number of relevant genes from 26 to 11. These 11 genes were *SERPINE1*, *MEF2B*, *S100Z*, *AXIN2*, *IGFBP1*, *GRP*, *ADH4*, *APOH*, *KRT15*, *ADTRP*, and *ADRA1B* (Figure 4F). After the final determination of the relevant genes, the risk scores of each sample in the training cohorts, based on their expression levels, were calculated and the patients were classified into high-risk or low-risk groups, according to the corresponding median scores. The overall survival (OS) rates of patients with high-risk scores were significantly lower than those with low-risk scores, indicating the association of high-risk scores with poor prognosis (Figure 4G). The performance of the prognostic prediction model was then analyzed using receiver operating characteristic (ROC) curves using the time ROC package. As shown in Figure 4H, the areas under the ROC curve (AUCs) were 0.71 (at 1 year), 0.77 (at 3 years), and 0.73 (at 5 years). The expression of the 11 signature genes was then verified using qRT-PCR (five matched clinical tissues of GC patients). The expression of these genes was found to be highly heterogeneous in GC samples (expression levels vary greatly across samples, Figure 4I). In addition to this, we also calculated the coefficient of variation for 5 matched clinical tissues of GC patients in cancerous and paraneoplastic tissues, which showed that these genes were more stably expressed in paraneoplastic tissues, but very unstably expressed in cancerous tissues (Figure 4J). These results further demonstrated the high heterogeneity of the expression of these genes in GC tissues.

### Risk models are associated with genomic instability

**FIGURE 5:**
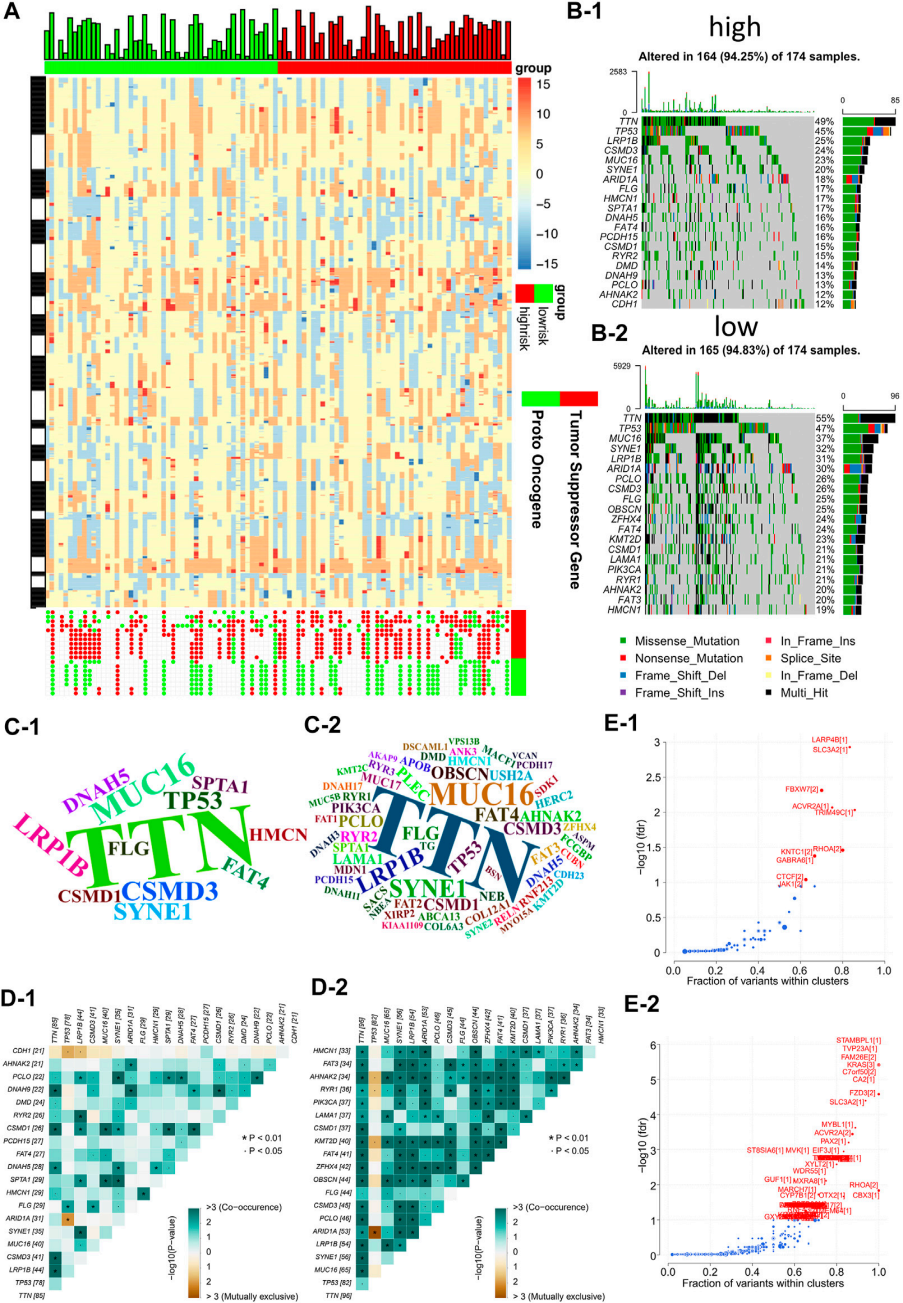
Risk models are associated with genomic instability **(A)** Comparing low and high risk group tumor sectors (top), bar chart showing the altered genome fraction (second top), heatmap showing differential copy number variations (CNV) level (middle) and copy number status of the selected genes showing significant deletion or amplification (bottom). **(B)** Waterfall plot of the top 20 mutant genes in the high and low risk groups (B-1: high risk group, B-2: low risk group). **(C)** Wordclouds were drawn with size of scripts bound up with amounts of samples containing certain mutant genes in the high and low risk groups (C-1: high risk group, C-2: low risk group). **(D)** Correlation heat map to show the degree of correlation between mutant genes in the high and low risk groups (D-1: high risk group, D-2: low risk group). **(E)** Volcano map used to demonstrate mutation driver genes in the high and low risk groups (E−1: high risk group, E−2: low risk group).

Firstly, the waterfall plot in Figure 5A depicts a variety of color annotations that distinguish distinct mutation types and the mutations associated with groups with different risk scores. It was found that missense mutations, single nucleotide polymorphisms (SNPs), and C > T mutations were most common in the different categories (Supplementary Figure S3A, B). The frequencies of variants in the different samples were then illustrated using boxplots, finding that frameshift mutations were more common in groups with high-risk scores, compared with groups with lower risk scores (Supplementary Figure S3C, D). As shown in Supplementary Figure S3E, F, there appeared to be differences in gene druggability between patients with different risks, and in the crosstalk between drugs and genes in these patient samples. Next, we investigated DNA copy number variations (CNVs) in tumors to elucidate whether the characteristics of our risk models can serve as signatures for tumor development. The results demonstrated that the incidence of CNVs was higher in groups with high-risk scores, compared with those with low-risk scores, indicating lower genome-wide stability and higher mutation loads in groups with high risks (Figures 5B–1, 2). In addition, word clouds were used with script sizes reflecting the frequencies of mutant genes in the different samples (Figures 5C–1, 2). The word clouds showed that the groups with low-risk scores harbored the greatest numbers of mutant genes, while groups with high risk scores contained the fewest. Additionally, we also analyzed the correlation between different mutations in the somatic cells of patients with different risks. This revealed that in the somatic cells of low-risk patients, the incidence of chain mutation and mutually exclusive mutations accounted for the most mutated genes, while in the high-risk patients, the incidence of co-mutations and mutually exclusive mutations accounted for the fewest mutated genes (Figures 5D–1, 2). This was consistent with the conclusion of Figure 3C and highlighted the relationship between the number of mutant genes and the prognosis of GC patients. Apart from the above, we also observed that patients with different risks possessed different numbers of driver mutations (Figures 5E–1, 2).

### Correlations between infiltration of tumor-associated immune cells and risk modeling

**FIGURE 6:**
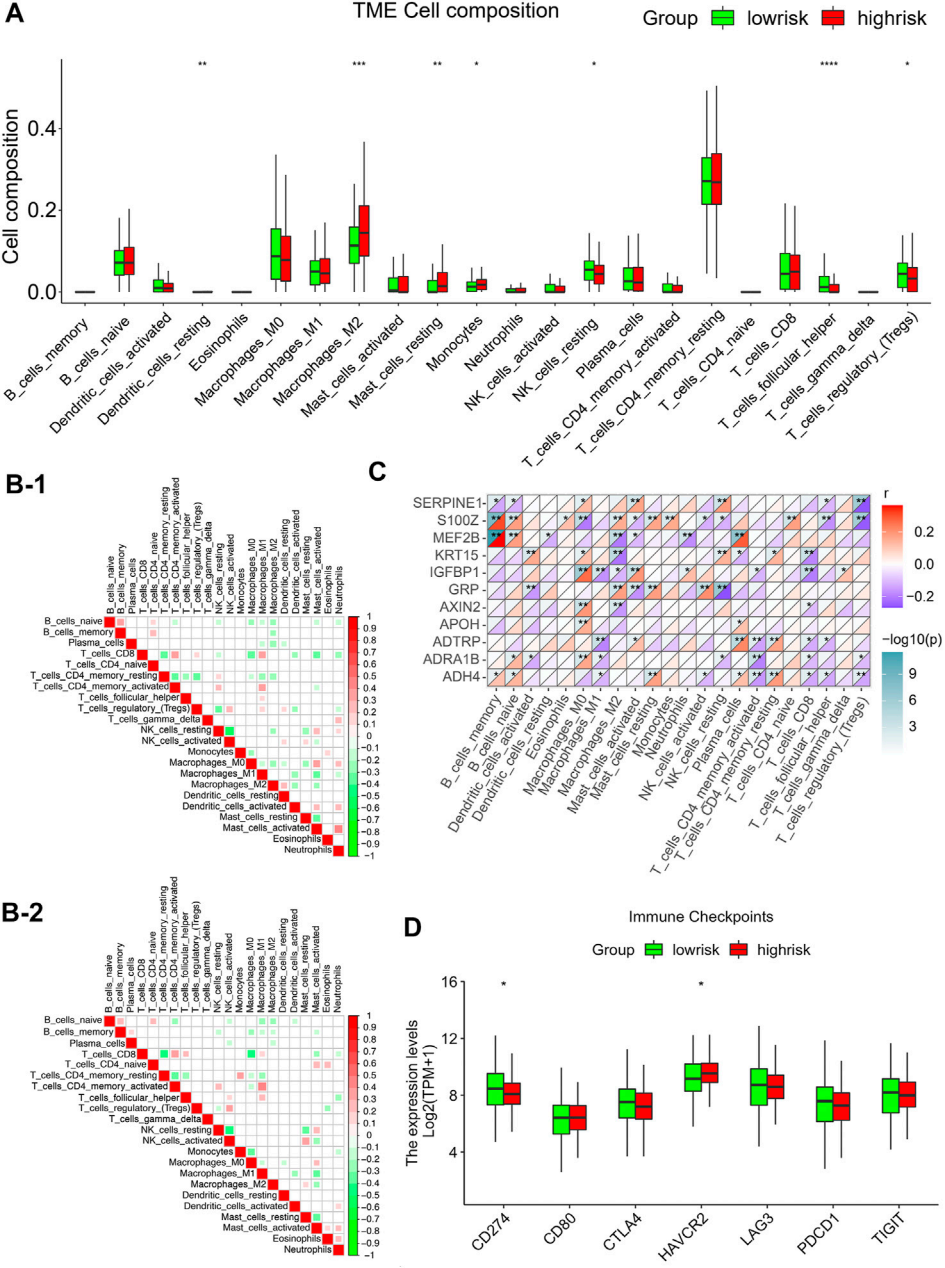
Correlations between infiltration of tumor-associated immune cells and risk modeling **(A)** Differential analysis of the level of infiltration of 22 immune cells in the high and low risk groups. **(B)** Different correlation patterns among 22 immune cell subsets in the high and low risk groups (B-1: high risk group, B-2: low risk group). **(C)** Heat map of the correlation between 11 gene signature and 22 immune cells. Red is used to represent the positive correlation between genes and immune cells, and blue is used to represent the *p*-value of the correlation between the two. (*p < 0.05; **p < 0.01; ***p < 0.001). **(D)** Expression of common immune checkpoints in high and low risk groups.

To identify the correlation between infiltration of immune cells and risk scores, the ESTIMATE-package was utilized to assess stromal, immune, and estimate scores. We first generated bar plots to show the proportions of 22 types of immune cells in GC samples (Supplementary Figure S4A). Next, we displayed the levels of these immune cells in samples with different risk scores using a heatmap (Supplementary Figure S4B). This showed marked differences in the infiltration scores of the immune cells between these samples, particularly, in terms of type Ⅱ macrophages, resting mastocytes, monocytes, resting NK cells, follicular helper T cells, and regulatory T cells (Figure 6A). Pearson correlations were analyzed using the cor. test function. Groups with high risks showed greater concentrations of M2 and mastocytes, implying deprivation of the immune microenvironment. To explore the tumor-associated immune cell crosstalk between high- and low-risk patients, a correlation heatmap of the 22 immune cell types was constructed. This showed differences in the immune microenvironments of groups with different risks (Figures 6B–1, 2). Meanwhile, we further probed the relationship between gene expression and infiltration of tumor-associated immune cells (TAICs) (Figure 6C). In addition, to ensure the reliability of the results, other immune scoring methods, namely, ssGSEA and MCP were also used to explore the differences in the immune microenvironment between the two groups (Supplementary Figure S4C, D). The results revealed that the two groups indeed had different microenvironments and immune cell interaction patterns. The levels of resting NK cells were positively associated with genes such as *MEF2B*, *APOH*, *ADTRP*, and *ADH4*, while the level of Tregs was negatively correlated with genes such as *SERPINE1*, *S100Z*, *ADRA1B*, and *ADH4*. Furthermore, we investigated the relationships between the risk models and immune checkpoints. The results showed that there were profound differences in *CD274* (PD-L1) and *HAVCR2* expression between the two groups (*p* < 0.05; Figure 6D). Importantly, the level of PD-L1 checkpoint was lower in the high-risk group, thus indicating that it is not appropriate to treat high-risk patients with PD-L1 inhibitors.

### Functional annotation and enrichment analyses of GC samples with different risks

**FIGURE 7:**
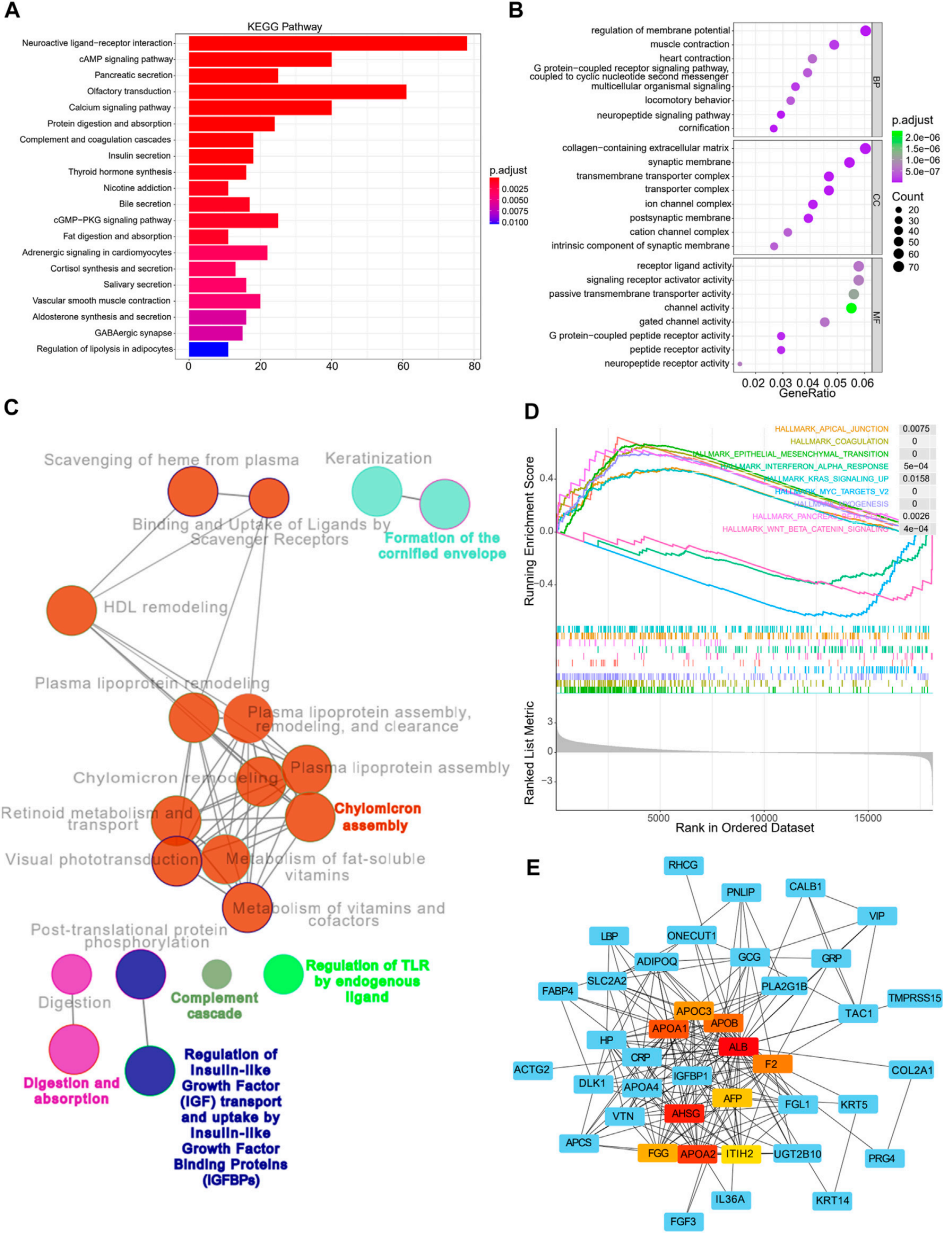
Functional annotation, genomic enrichment analysis of gastric cancer samples with different risks **(A)** KEGG enrichment analysis shows the top 20 pathways in high and low risk groups. **(B)** Dot plot shows enriched GO terms of upregulated 11 gene signature. **(C)** Functional access network shows transcriptome profiles of different risk groups. **(D)** GSEA enrichment analysis shows the 9 hallmarks gene sets in the high and low risk groups. **(E)** The protein–protein interaction network of the high and low risk groups.

Enrichment analyses were then performed to investigate the underlying biological processes that affect the risk scores. Using the TCGA cohorts, 1,183 upregulated and 138 downregulated genes were identified between the high and low-risk subgroups (with a threshold of *p* < 0.05 and absolute of |log_2_ FC|> 2, Supplementary Figure S5A). KEGG and GO analyses indicated that these genes were primarily enriched in pathways such as neuroactive ligand-receptor interaction, cAMP signaling pathway, pancreatic secretion, olfactory transduction, and calcium signaling pathway, amongst others (Figure 7A). In addition, GO annotations revealed enrichment in the regulation of membrane potential, collagen-containing extracellular matrix, receptor-ligand activity, and signaling receptor activator activity, amongst others (Figure 7B). The functional networks between the DEGs were constructed using Cytoscape, and the potential biological networks affecting risk scores are shown in Figure 7C. Consistent with these findings, the results from GSEA demonstrated that samples with high risks were correlated with activation of typical carcinogenic characteristics, involving the EMT and the KRAS and MYC pathways, suggesting the underlying mechanisms associated with poor prognosis in the high-risk samples (Figure 7D). Furthermore, we constructed a gene-related network based on the risk scores-related DEGs. This showed that the expression of genes such as *ALB*, *F2*, *APOA1*, *APOA2*, *APOA3*, and *AHSG* was affected by risk scores (Figure 7E). The expression of these genes in terms of high and low-risk scores in tumor and normal tissues are shown in Supplementary Figure S5C, while the correlations between these genes and patient prognosis are shown in Supplementary Figure S5D.

### Risk model validation and nomogram construction

**FIGURE 8:**
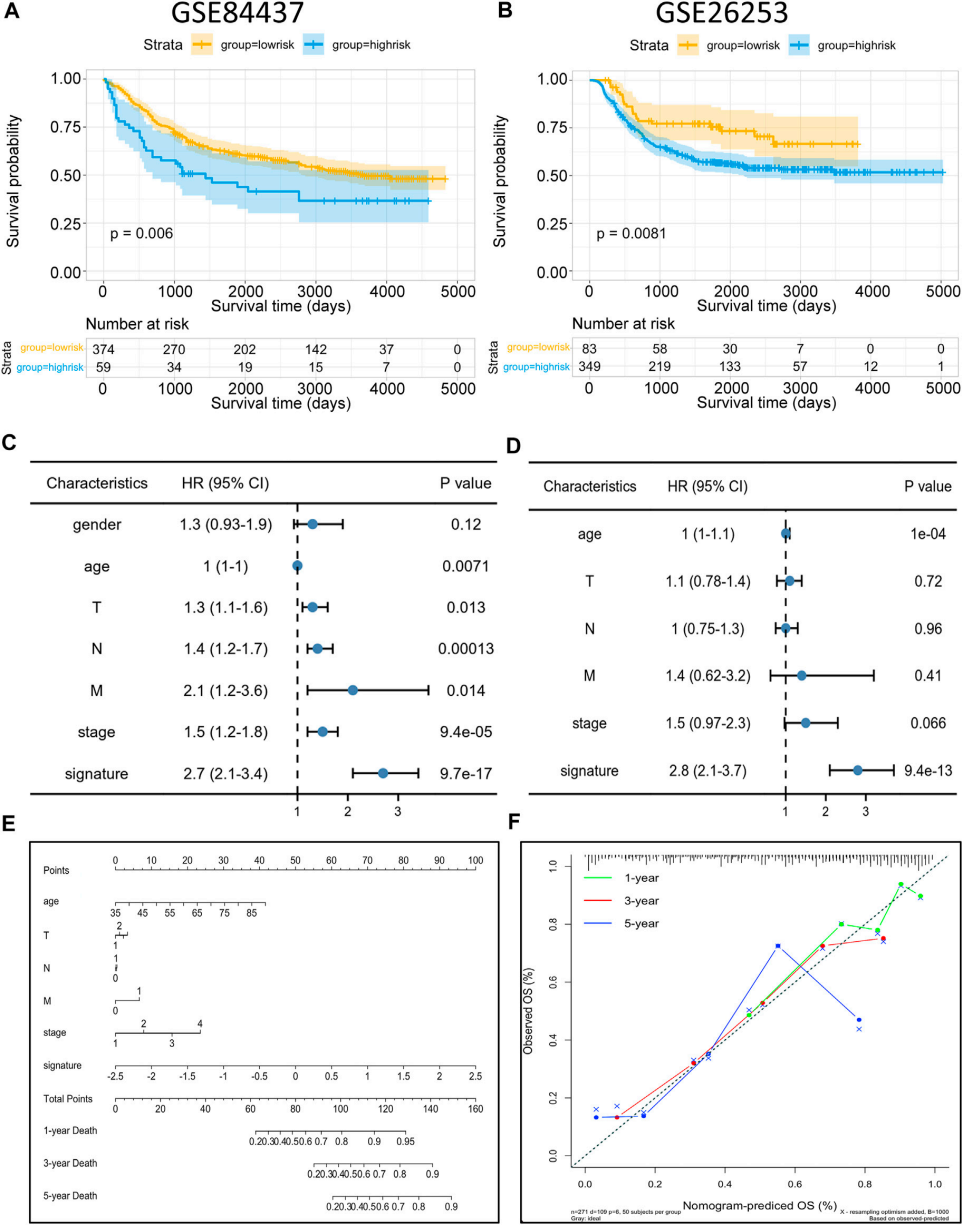
Risk model validation and nomogram construction (**A,B**) Kaplan–Meier OS curves with difference detection by log-rank test for patients from the validation datasets (GSE84437 and GSE26253). (**C,D**) Multivariate analysis of clinical and biological variables using Cox proportional hazards regression models, suggesting that 11 gene signature is the independent risk factors of prognosis of patients. (**E**) Composite nomogram prediction of 1-year, 3-year, and 5-year OS. (**F**) Calibration curves of observed and predicted probabilities for the nomogram.

To identify the robustness of the model, the same models and coefficients from the training cohorts were applied to the external cohorts. The risk scores of each sample were calculated according to their gene expression, and the distributions of the risk scores were analyzed. In the independent validation cohort GSE84437, the best cutoff point was employed for grouping. As shown in Figure 8A, the prognosis of high-risk patients (59 samples) was significantly worse than that of low-risk patients (374 samples) (*p* = 0.006). Meanwhile, the same analysis was conducted with the independent validation cohort GSE26253, and resulted in the same conclusion, i.e., the prognosis of the high-risk patients (349 samples) was significantly worse than that of low-risk patients (83 samples) (*p* = 0.0081; Figure 8B), thus, confirming the reliability of the model. The risk scores were then incorporated with the corresponding clinical characteristics, and the results from the univariate Cox regression analyses revealed a strong correlation between the risk scores and GC patient prognosis (Figure 8C). Multivariate Cox regression analysis further revealed that the risk score (*p* < 9.4e-13, HR = 2.8, 95%CI2.1–3.7) can serve as an independent risk factor for GC patient prognosis (Figure 8D). Interestingly, the multivariate analysis, in conjunction with the signature, showed that the originally meaningful TNM staging was no longer significant (*p* > 0.05). This suggests that the association between TNM staging and patient prognosis was influenced by metabolic signature, i.e., the effect of TNM staging on tumor prognosis may be acting through the metabolic signature. Apart from this, we also constructed a nomogram using the most essential variables in the multivariate analyses (Figure 8E). The nomogram showed that the risk score had the greatest impact on GC patient prognosis. This indicated that the risk model, based on 11 signature genes, was highly effective for prognostic prediction. Lastly, results from the calibration curve analyses showed that the calibration curve for prediction closely resembled the standard curves of survival at the 1-, 3-, and 5-year follow-ups, confirming the above conclusion (Figure 8F). Compared to nomograms for GC prognosis developed in previous studies, the present model was more accurate for prognosis prediction at the 1 and 3-year time points but, unfortunately, had worse predictive efficacy at the 5-year time point (Cai et al., 2020).

## Discussion

Metabolic reprogramming promotes tumor occurrence and progression (Boroughs and DeBerardinis, 2015; Pavlova and Thompson, 2016). Metabolic phenotypes can aid in tumor imaging, prognosis determination, and cancer treatment (Vander Heiden and DeBerardinis, 2017). However, the application of tumor metabolism as one an indicator of clinical behavior requires clarification of the metabolic pathways that restrict cancer progression, as well as ascertaining the metabolic characteristics and heterogeneity of each patient with GC (Li et al., 2019).

While GC is known to be a highly heterogeneous disease, few studies have investigated its specific molecular subtypes. Zhang et al. (2016) reported 53 mutations in the microRNA-related molecular subtypes of GC but did not investigate the functional characteristics associated with these subtypes. Yang et al. (2021) described subtypes associated with high microsatellite instability but did not use multi-omics analysis, which is more meaningful for risk stratification of cancer patients. Jiang et al. reported ferroptosis-related molecular subtypes but did not further investigate the microenvironmental and genomic differences of these subtypes (Yang et al., 2021). Metabolism-related subtypes have been reported in other tumors, such as breast and colon cancer, but no metabolism-related molecular subtypes were previously reported in GC (Chong et al., 2021; Gong et al., 2021). In 2021, Gong et al. (2021) reported on the identification of metabolic subtypes of triple negative breast cancer, based on the core metabolic pathways of lipid metabolism, glycolysis, and nucleic acid metabolism. This demonstrated the metabolic heterogeneity of breast cancer.

Metabolic heterogeneity is one of the future trends in tumor research. However, there are reports on the metabolic heterogeneity of GC based on molecular typing (Kim and DeBerardinis, 2019; Martinez-Reyes and Chandel, 2021). In our study, we comprehensively analyzed core metabolic activities related to signature genes in GC samples. This helped broaden our insight into the metabolic characteristics and heterogeneity of each GC patient. Hence, it is essential to emphasize the distinctions between metabolic adaptations in individual GC patients. To begin our research, we clustered the patients into three subtypes, based on their expression of GSEA-signature genes related to various metabolic pathways, including glycolysis, lipid metabolism, amino acid metabolism, and nucleic acid metabolism. We observed that subtype-1 patients showed the best prognosis, while the other two subtypes had worse prognosis. Besides, we also discovered significant differences in genomic characteristics among the three subtypes and identified a new driver gene, *CNBD1*, that drove mutations in GC. The subtypes were ranked as NMF1 to NMF2 to NMF3 in terms of increasingly poor prognosis, and the evolutionary driver genes for the three subtypes also showed the same pattern of variation. This observation is very interesting and suggests that the worse the patient prognosis, the greater the evolutionary pressure and, therefore, the less the expression of evolutionary driver genes.

Most investigations into tumor metabolism have focused on a specific metabolic process in the patients (Huang et al., 2020; Xu et al., 2020), thus, ignoring the differences in combinations of metabolic preferences in each GC patient. For instance, a seven-gene signature was established, based only on glycolysis (Yu et al., 2020), and Zhou Zhu et al. identified six GC subtypes according to the cholesterol metabolism and glycolytic pathways (Zhu et al., 2021b). Apart from these, signatures based on lipid metabolism pathways have been shown to play important roles in the prognosis prediction of GC patients (Hu et al., 2020). In this study, we established three subtypes based on the distinct activity of core metabolic processes such as glycolysis, lipid metabolism, amino acid metabolism, and nucleic acid metabolism in GC samples. Of these three subtypes, subtype 1 was associated with good prognosis, while the remaining two subtypes were linked with poor prognosis. The differences between the poor-prognosis subtypes were apparent after two years, when it was clear that subtype 3 offered the worst outcome.

Tumors are complex regulatory systems, and the limitations of studies based on a single set of histological data are apparent. Thus, integrated analysis of high-throughput, multi-omics data, using multiple levels and sources, is imperative. Moreover, like other investigators, we also investigated the potential of the three metabolic subtypes in terms of genomics (Su et al., 2020). Our results were truly interesting in that they revealed that driver genes were more common in subtype 1 although subtype 1 was associated with the best prognosis. In contrast, subtype 3 patients experienced the worst prognosis, while having only one driver gene, *CNBD1*, with subtype 2 falling between subtypes 1 and 3 in both aspects. According to Li Qiangchun et al.’s work, CNBD1 drives a high incidence of mutations, including non-synonymous mutations, in genes, which is relevant to the prognosis of GC patients (Li et al., 2016). However, we demonstrated the role of *CNBD1* in GC, where it functions as an evolutionary driver gene, resulting in metabolic changes leading to malignant changes and poor prognosis in GC patients. In addition, we also demonstrated that the patients with more mutations exhibited better prognosis while patients with fewer mutations had the opposite outcome, which is consistent with the results from *JAMA Oncology* (Li et al., 2018). *PRIM1* may be associated with this mutation pattern (Zhu et al., 2021c). Furthermore, we examined the correlations between mutations in the somatic cells of the three subtypes. This showed that patients with more chain mutation and mutually exclusive mutations had a better prognosis, highlighting the relevance of the signatures in GC patient prognosis. Subsequently, we identified 412 prognosis-related DEGs in the different metabolic subtypes using gap analyses and univariate Cox regression. These genes were then incorporated into LASSO regression analysis and a random forest model, leading to the selection of 11 genes in the metabolism-related prognostic signature, including *SERPINE1*, *MEF2B*, *S100Z*, *AXIN2*, *IGFBP1*, *GRP*, *ADH4*, *APOH*, *KRT15*, *ADTRP*, and *ADRA1B*. The model based on these genes was found to be both effective and robust in different patient cohorts, and multivariate Cox regression showed that the model could serve as an independent predictor of prognosis in GC patients. Additionally, we constructed a nomogram for the prediction of patient OS, and the efficacy of the nomogram was further confirmed using calibration curve analyses. In terms of the KEGG enrichment analysis, the 11 signature genes were found to be mainly enriched in pathways associated with neuroactive ligand-receptor interaction, cAMP signaling, pancreatic secretion, olfactory transduction, and calcium signaling. Moreover, GO analysis showed enrichment in the regulation of membrane potential, collagen-containing extracellular matrix, receptor-ligand activity, and signaling receptor activator activity, amongst others (Kanehisa and Goto, 2000).

Furthermore, a variety of cells, cytokines, exosomes, and chemotactic factors are able to interact and communicate with cancer cells in the TME, especially TAICs which display anti-tumor functions (Gajewski et al., 2013; Hinshaw and Shevde, 2019; Zhang et al., 2021). TME fibroblasts are recognized as tumor targets and play an integral role in tumor growth and metastasis (Zhang et al., 2020b). In our study, we observed significant differences in the proportions of infiltrating immune cell types between patients with high and low-risk scores, specifically, type Ⅱ macrophages, resting mastocytes, monocytes, resting NK cells, follicular helper T cells, and regulatory T cells. This was consistent with the subsequent finding that the immune microenvironment differed in groups with different risk scores. High-risk groups showed greater concentrations of M2 macrophages and mastocytes, suggesting depletion of the immune microenvironment (Liu et al., 2011; Liao et al., 2018). We also explored the correlation between TAIC infiltration and the expression of risk model-related genes. The results showed that the proportion of resting NK cells was positively associated with genes such as *MEF2B*, *APOH*, *ADTRP*, and *ADH4*, while the Treg level was negatively correlated with *SERPINE1*, *S100Z*, *ADRA1B*, and *ADH4*. This is consistent with the findings of other studies (Feske et al., 2015; Chu et al., 2020; Huang et al., 2021). Furthermore, we investigated the relationships between the risk models and immune checkpoints, finding significant differences in the levels of *CD274* (PD-L1) and *HAVCR2* between high- and low-risk patients. Notably, the level of PD-L1 was lower in the high-risk patients, suggesting that high-risk patients should not be treated with PD-L1 inhibitors. Additional investigations into the precise mechanisms of tumor-associated immune cells and their association with GC prognosis are still required in the future.

## Conclusion

Taken together, we identified three GC subtypes based on core metabolic pathways and constructed a prognostic model using 11 signature genes that were differentially expressed in the three metabolic subtypes. The model was found to be both effective and robust in different patient cohorts and was shown by multivariate Cox regression to be an independent predictor of GC patient prognosis. We also investigated the genomic characteristics of the three subtypes, as well as our models, observing an association between GC-associated immune cells and the 11 signature genes.

## Data Availability

The datasets presented in this study can be found in online repositories. The names of the repository/repositories and accession number(s) can be found in the article/Supplementary Material.
