# Supplementary material for: Identification of three metabolic subtypes in gastric cancer and the construction of a metabolic pathway-based risk model that predicts the overall survival of GC patients
Source: Front Genet. 2023 Feb 10;14:1094838. doi: 10.3389/fgene.2023.1094838 (PMC9950121; doi:10.3389/fgene.2023.1094838)
Supplement: Supplementary file 1 [file DataSheet2.PDF]

#*Supplementary Material*

Supplementary Tables

Supplementary Table 1 mRNA PCR primer

Gene name	Sequence of primer
SERPINE1	F: CCTGGGCACTTACAGGAAGG
	R: GGTCCGATTCGTCGTCAAATAAC
MEF2B	F: CCTGGACCAAAGGAATCGGC
	R: TGAGGGCTATCTCACAGTCAC
S100Z	F: TCTTCCACCGCTATTCTGGC
	R: TCCAGGTCCTGCACTATCTTATC
AXIN2	F: CAACACCAGGCGGAACGAA
	R: GCCCAATAAGGAGTGTAAGGACT
IGFBP1	F: TTGGGACGCCATCAGTACCTA
	R: TTGGCTAAACTCTCTACGACTCT
GRP	F: ACAATCCCGGAAGGACAACTC
	R: GTCTATGTCGATTAGGCTGCTG
ADH4	F: AGTTCGCATTCAGATCATTGCT
	R: CTGGCCCAATACTTTCCACAA
APOH	F: CCCAAGCCAGATGATTTACCAT
	R: ACAGTCCTGTGAGAGGGCA
KRT15	F: TCTGCTAGGTTTGTCTCTTCAGG
	R: CCAGGGCACGTACCTTGTC
ADTRP	F: GTGCTGAAAAGAACCAAAGGGG
	R: AGGACCTTGGGGTAAATGAGAT
ADRA1B	F: TGGGGCGGATCTTCTGTGA
	R: GTGACCAGCGTGGGATACTG
β -actin	F:CGTGATGGTGGGCATGGGTCAG
	R:CTTAATGTCACGCACGATTTCC

Supplementary Figures

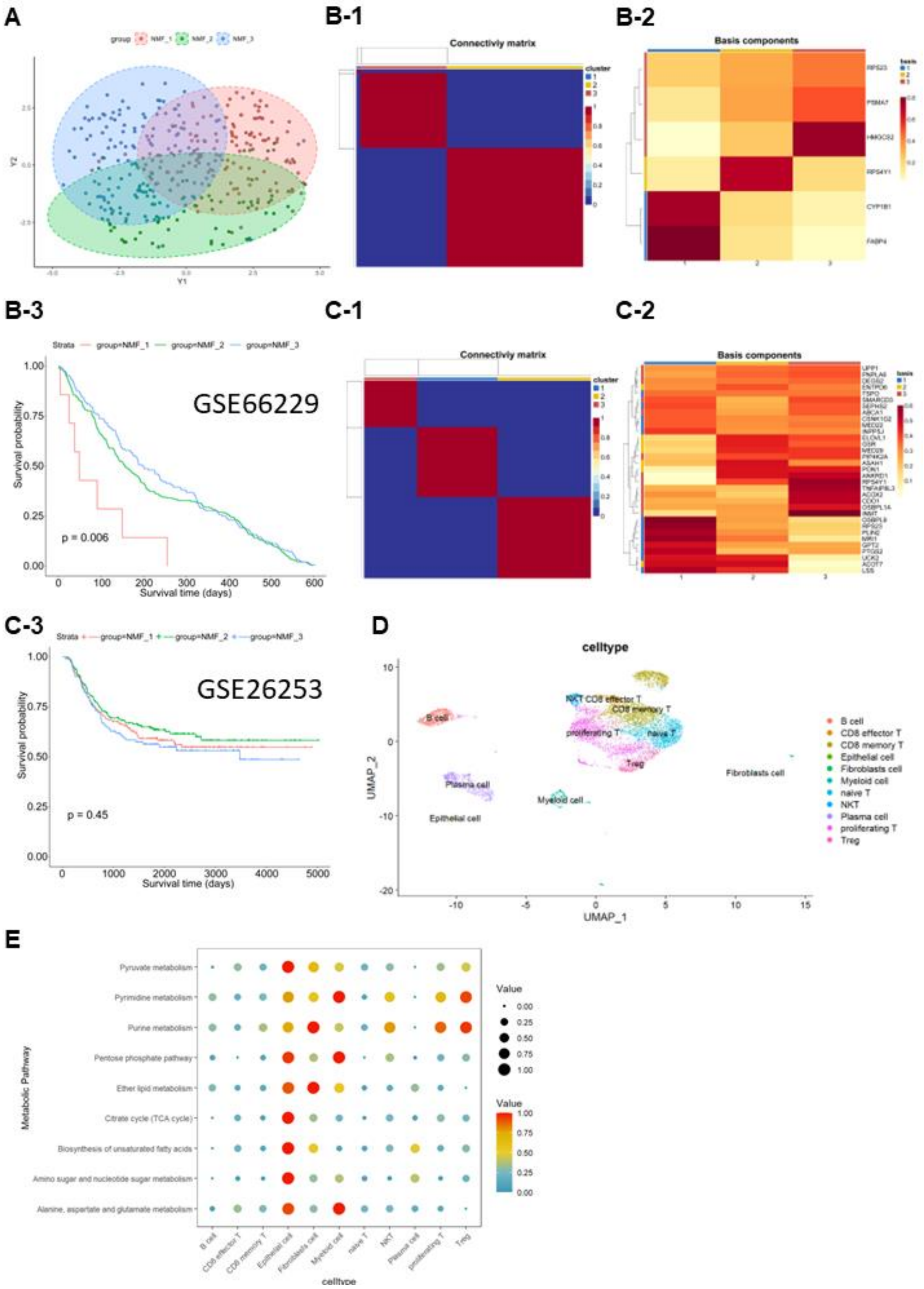

Supplementary Figure 1. (**A**) These three subtypes could be distinguished by means of PCA and clustering. (**B-1**) GC samples from GSE66229 were clustered by nonnegative matrix factorization (NMF) method. (**B-2**) Classification of expression profiles into 3 categories using NMF (GSE66229). （**B-3**）Survival analysis was used to evaluate the different survival patterns between metabolic subtypes (GSE66229). (**C-1**) GC samples from GSE26253 were clustered by

nonnegative matrix factorization (NMF) method. (**C-2**) Classification of expression profiles into 3 categories based on signature levels using NMF (GSE26253). （**C-3**）Survival analysis was used to evaluate the different survival patterns between metabolic subtypes (GSE26253). (**D**) Single UMAP identifies major cell populations in tumor tissue, while (**E**) represents cluster analysis of core metabolic pathways across multiple cell types.

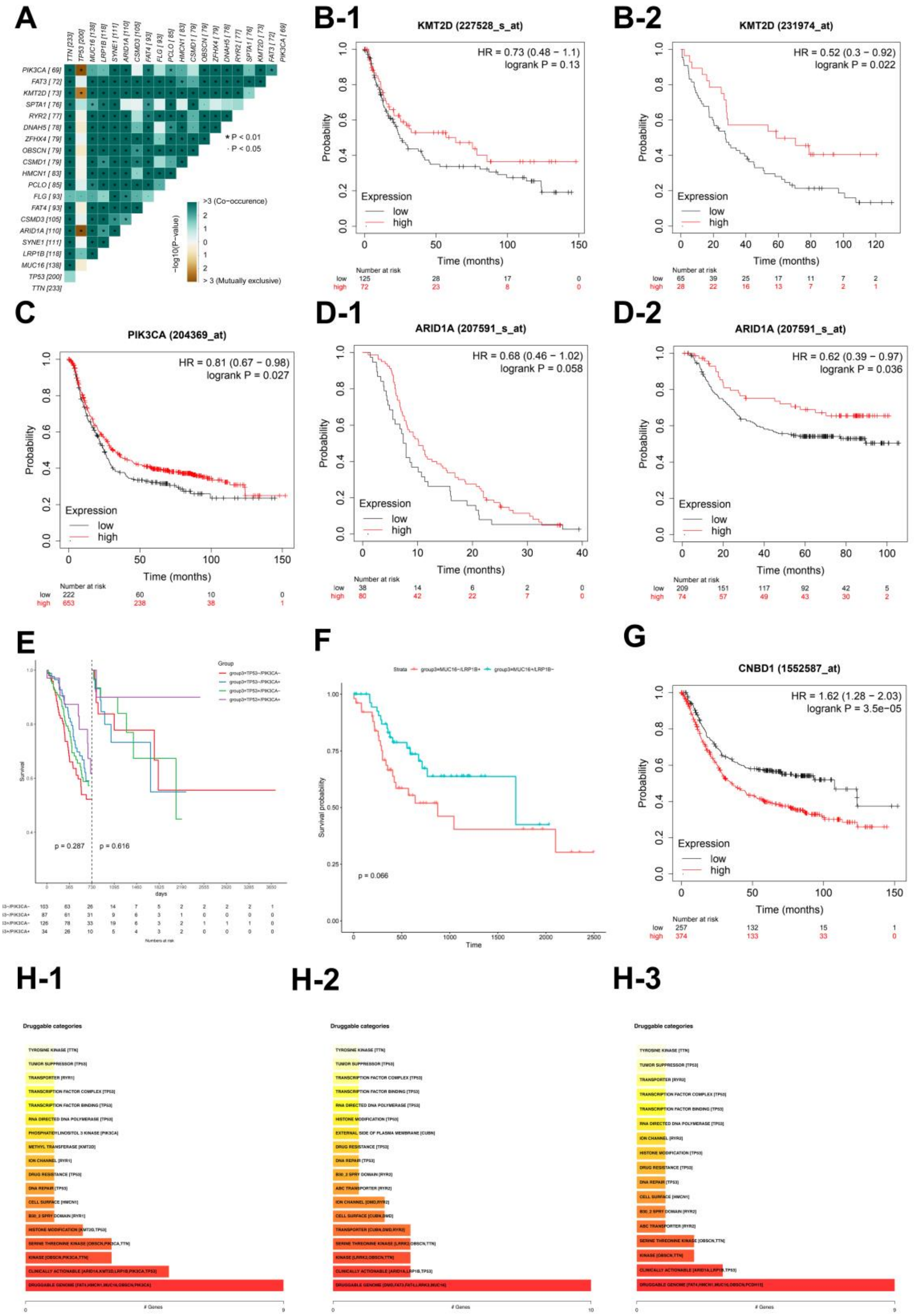

Supplementary Figure 2. (**A**) Correlation heat map to show the degree of correlation between mutant genes in all 3 metabolic subtypes. (**B**) Kaplan-Meier graphs showing KMT2D low (black) or high (red); Hazard ratio (HR) and log-rank test two-tailed P-values as indicated. (**C**) Kaplan-Meier graphs showing PIK3CA low (black) or high (red); Hazard ratio (HR) and log-rank test two-tailed P-values as indicated. (**D**) Kaplan-Meier graphs showing ARID1A low (black) or high (red); Hazard ratio (HR) and log-rank test two-tailed P-values as indicated. (**E**) Survival analysis was used to evaluate the different survival patterns of TP53-/PIK3CA-, TP53-/PIK3CA+, TP53+/PIK3CA-, and TP53+/PIK3CA+ patients. (**F**) Survival analysis was used to evaluate the different survival patterns between MUC16-/LRP1B+ and MUC16+/LRP1B- patients. (**G**) Kaplan-Meier graphs showing CNBD1 low (black) or high (red); Hazard ratio (HR) and log-rank test two-tailed P-values as indicated. (**H**) The bar chart shows druggability of mutant genes in these subtypes and the crosstalk between genes and drugs (H-1: NMF_1, H-2: NMF_2, H-3: NMF_3).

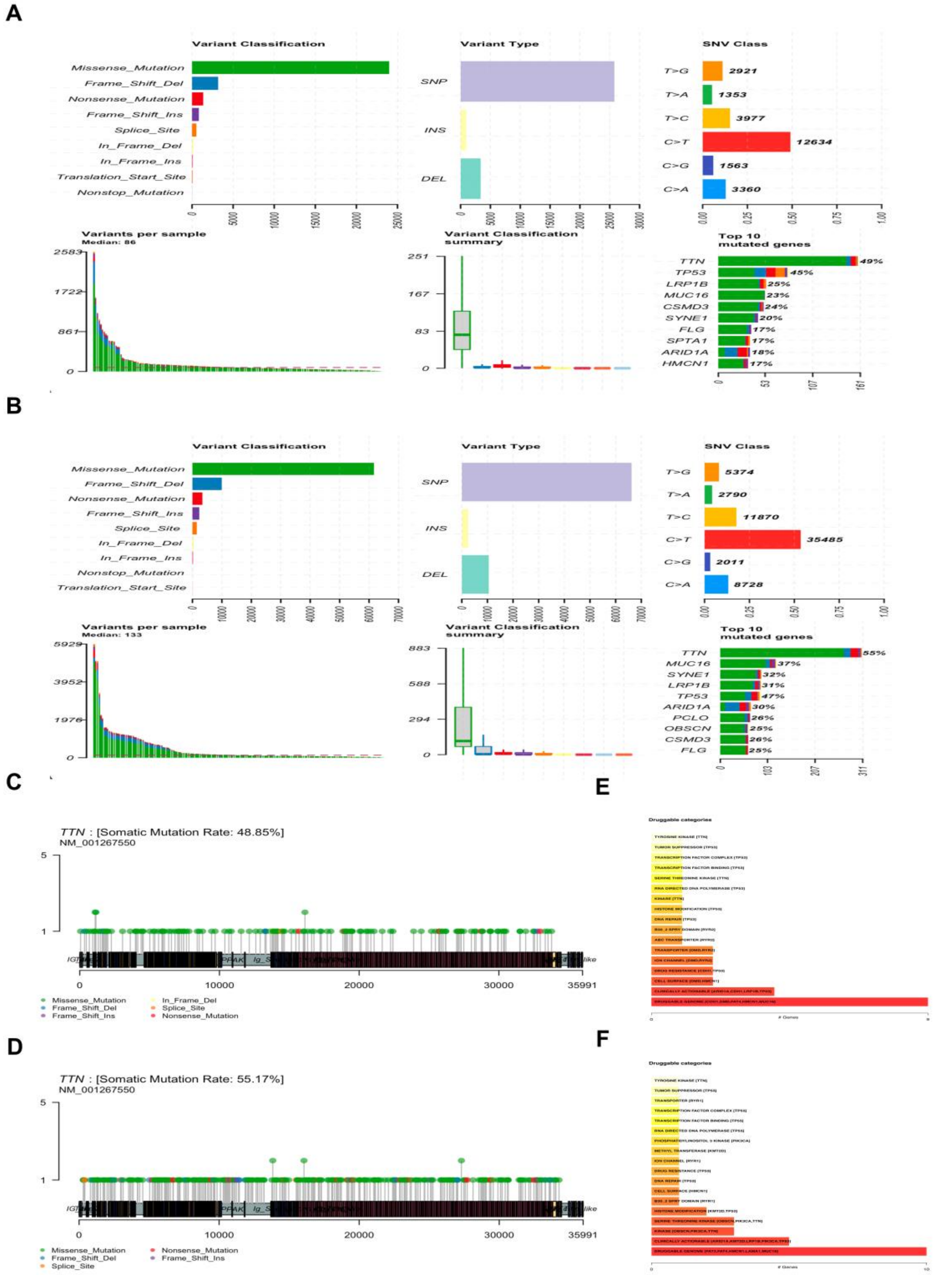

Supplementary Figure 3. (**A-B**) Display of single nucleotide polymorphisms (SNPS) in the high and low risk groups. (**C-D**) Differences in TNN gene mutation sites in the high and low risk groups. (**E-F**) The bar chart shows druggability of mutant genes in the high and low risk groups and the crosstalk between genes and drugs.

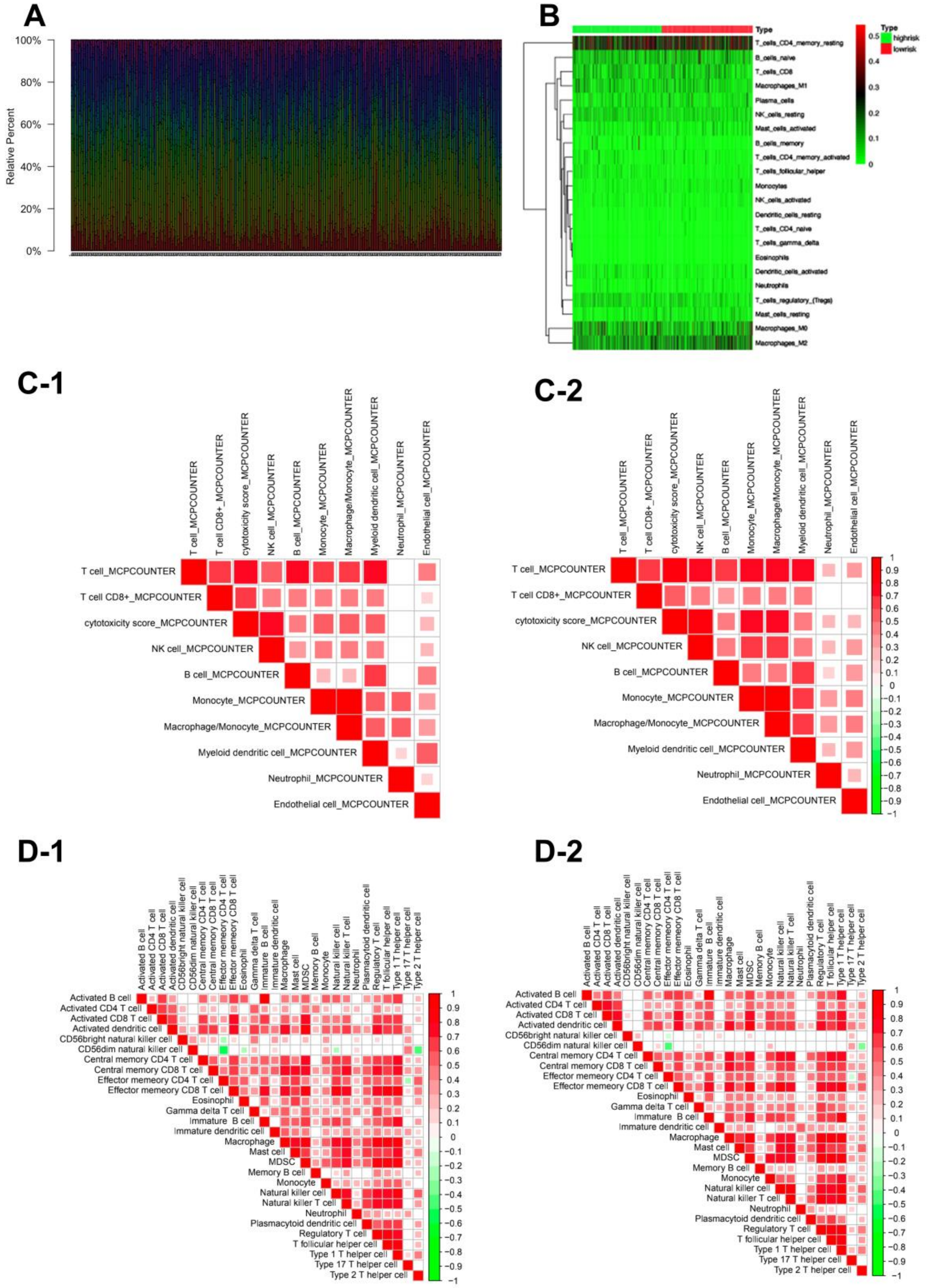

Supplementary Figure 4. (**A**) Bar plots was exhibited a ratio of 22 types of immune cells in gastric cancer samples. (**B**) Heatmap of those immune cells in samples with distinct risk scores. (**C**) Different correlation patterns among immune cell subsets in the high and low risk groups by MCP (C-1: high risk group, C-2: low risk group). (**D**) Different correlation patterns among immune cell subsets in the high and low risk groups by ssGSEA (D-1: high risk group,D-2: low

risk group).

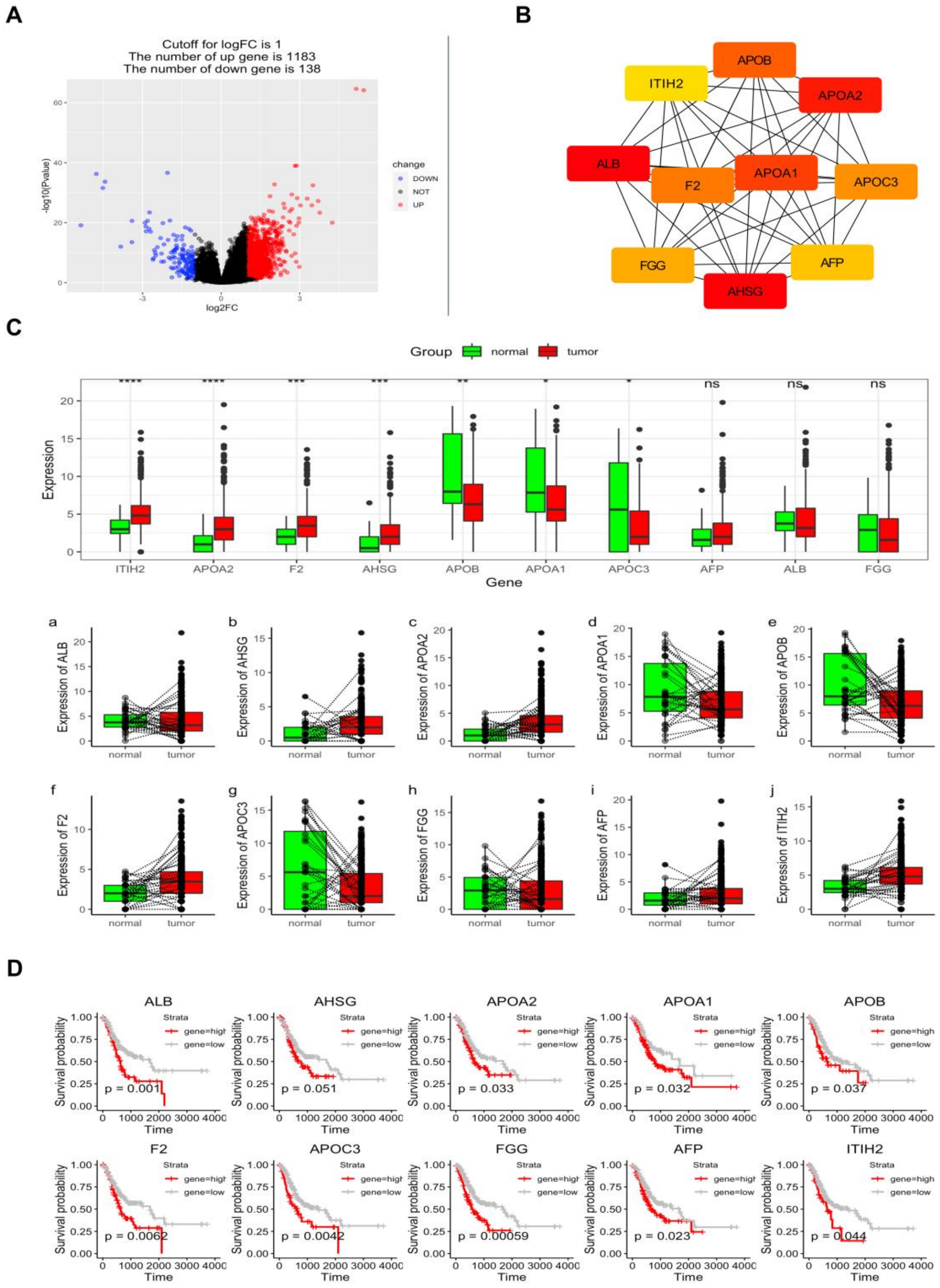

Supplementary Figure 5. (**A**) Volcano map showing differential genes between high and low risk groups. (**B**) Top 10 genes in the protein-protein interaction network in the high-risk and low-risk groups. (**C**) Expression levels of top 10 genes in gastric cancer and paraneoplastic tissues. (**D**) Kaplan–Meier OS curves with top 10 genes by log-rank test for gastric cancer patients.
